# Congenital tuberculosis detected by T-SPOT.TB assay in a male infant after *in vitro* fertilization and followed up with radiography

**DOI:** 10.1186/s13052-014-0096-0

**Published:** 2014-11-27

**Authors:** Yangming Zheng, Guanghui Bai, Hailin Zhang

**Affiliations:** Department of Pediatric Pulmonology, The Second Affiliated Hospital & Yuying Children’s Hospital, Wenzhou Medical University, No. 109, Xueyuan Xi Road, Wenzhou, Zhejiang PR China; Department of Radiology, The Second Affiliated Hospital & Yuying Children’s Hospital, Wenzhou Medical University, Wenzhou, Zhejiang PR China

## Abstract

Congenital tuberculosis (TB) is a rare disease with a high mortality rate, and is difficult to diagnose. Here we present a case of congenital TB detected by the T-SPOT.TB assay in a male infant after *in vitro* fertilization. He ultimately survived after anti-TB therapy despite a delayed diagnosis, and underwent radiological follow-up. The delay in diagnosis of congenital TB resulted in a severe lung lesion, as evidenced by prolonged oxygen dependence, predisposing to recurrent pneumonia. Radiological follow-up revealed uniform rim calcification of multiple enlarged lymph nodes in the mediastinum, and long-term consolidation in the bilateral lung, with slow radiographic regression of the lung lesion. To the best of our knowledge, this is the first report on using the T-SPOT.TB assay in the detection of congenital TB, and no case of congenital TB with such clinical features and image findings has been described in previous reports.

## Background

Congenital tuberculosis (TB) occurs in infants infected with *Mycobacterium tuberculosis* (MTB) *in utero* or during delivery. The infants may be infected by hematogenous dissemination of MTB from the maternal circulation, or by swallowing and aspirating infected amniotic fluid or maternal blood [1]. The disease can quickly progress to death if not treated promptly. Early diagnosis of congenital TB is essential for survival of the infected infant, but poses a great challenge because of non-specific symptoms that mimic other common neonatal illnesses. Tuberculin skin tests (TSTs) are also usually unreliable in younger infants [2,3].

Around 350 cases of congenital TB with varied symptoms have been reported to date. However, using the T-SPOT.TB assay in the detection of congenital TB has never been reported. Cases of congenital TB with prolonged oxygen dependence predisposing to recurrent pneumonia have not been described in the literature. In addition, as far as we are aware, there has been no report regarding serial radiological follow-up of congenital TB after anti-TB therapy.

## Case presentation

A male infant was admitted to neonatal care after birth because of prematurity. He was born to a 35-year-old Chinese woman, who conceived after *in vitro* fertilization. The infant was vaginally delivered at a gestation of 34 weeks, with obstetric risk from prolonged ruptured membranes. He weighed 2300 g at birth, and neonatal assessment was consistent with gestational age. Physical examination at admission was unremarkable, and laboratory tests were normal. He was vaccinated with the BCG vaccine. Because of possible infection by prolonged membrane rupture, penicillin was administered after admission. The patient progressed well during hospitalization, and he was discharged at 16 days of age.

Three days after discharge, his temperature rose to 38.7°C, and after 2 days of fever, he was readmitted to hospital. On examination, the patient was febrile to the touch with a fever of 38.5°C, but the rest of the examination was unremarkable. Blood investigations revealed a white blood cell count of 11,700/μL with a shift to the left, a C-reactive protein level of 24.7 mg/L (normal <8 mg/L), a total bilirubin level of 35 μmol/L, and an indirect bilirubin level of 23 μmol/L. Levels of aspartate aminotransferase and alanine aminotransferase were normal. Cerebrospinal fluid (CSF) was normal. Blood and CSF cultures were sterile. Serology was negative for HIV and syphilis. Screening for TORCH infection was negative.

**Figure 1:**
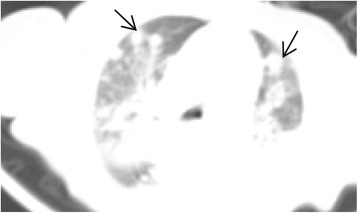
**Chest CT.** Multiple lung nodules (black arrows) and dense consolidation of the perihilar region were seen on the 19th hospital day. CT, Computed tomography.

The patient was initially started on cefoperazone-sulbactam and penicillin for presumed sepsis but the fever persisted, and a nonproductive cough and respiratory distress developed after admission. A chest radiograph performed on the 7th hospital day showed bilateral interstitial and alveolar infiltrates, which obscured the right heart, and a diagnosis of pneumonia was made. The antibiotics were changed to imipenem-cilastatin and vancomycin after the patient’s condition worsened, but there was no improvement. Non-enhanced computed tomography (CT) of the thorax was performed on the 19th hospital day, and revealed multiple lung nodules, with dense consolidation of the perihilar region (Figure 1). The condition of the patient continued to deteriorate.

On the 40th day after admission, respiratory failure occurred and the infant was placed on mechanical ventilation, when hepatosplenomegaly was noted. A test using the T-SPOT.TB assay (Immunotec, Oxford, UK) was performed on peripheral blood mononuclear cells, which were separated by centrifugation from a 4-mL blood sample obtained from the femoral vein. Spot-forming units (SFUs) were counted by an automated enzyme-linked immunospot (ELISPOT) reader (AID, Strassberg, Germany). SFU values in the negative-control well (SFU <5) were subtracted from SFU values in wells containing MTB-specific antigens ESAT-6 or CFP-10, with positive results (15 SFU for ESAT-6, 18 SFU for CFP-10). Although smears of endotracheal aspirates were negative for acid-fast bacilli (AFB), culture of endotracheal aspirates was pending. A presumptive diagnosis of TB was made.

Anti-TB drugs and methylprednisolone were prescribed on hospital day 49, and the patient became afebrile the following day. Culture of endotracheal aspirates yielded MTB in 6 weeks, which confirmed the diagnosis. Healthcare workers and family members were evaluated as possible sources of transmission in the infected infant. Healthcare workers were evaluated by chest radiographs and TSTs, but no pulmonary source of TB in the infant was found. The infant was separated from his parents and family members because of hospitalization after birth. The parents were the only postnatal family contacts with the infant during the 3 days before the onset of fever. His father was healthy and had a normal chest radiograph, though a TST showed an induration of 15 mm at 48 h. The patient’s mother had a history of consulting a gynecologist for infertility. She was free of symptoms apart from a self-limited episode of low-grade fever when she was 2 months’ pregnant. Her chest CT showed fibronodular lesions with calcifications in the bilateral upper lung, and her TST result was positive, with an induration of 18 mm at 48 h; smears of her sputum were negative for AFB.

It was unlikely that the parents were the source of postnatal transmission via the respiratory tract because they had no evidence of active pulmonary TB. Moreover, the infant had lived with his parents for only 3 days before the onset of fever, which was too short a time for the development of active TB. The patient was not breast-fed, thus infection via breast milk was also excluded.

Postnatal infection of the infant was ruled out after thorough contact investigations, and he was diagnosed with congenital TB. The condition of the patient gradually improved after anti-TB therapy; mechanical ventilation was stopped on hospital day 93, and he was discharged on intermittent home oxygen therapy on hospital day 117.

**Figure 2:**
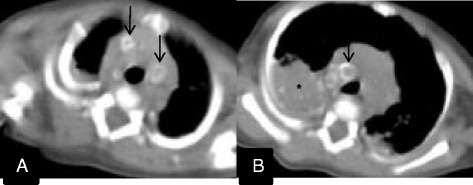
**Follow-up chest CT at 5 months of age. A**: There was uniform rim calcification of multiple enlarged lymph nodes in the mediastinum (black arrows). **B**: Dense mass-like consolidation of the bilateral lower lobe of the lungs remained, with multiple fine spots of calcification (star).

**Figure 3:**
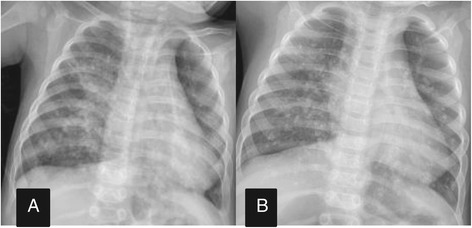
**Chest radiography. A**: A follow-up chest radiograph showed opacities in the bilateral lung remaining at 12 months of age. **B**: Chest radiography at 15 months showed nearly complete resolution of opacities with residual extensive spots of calcification.

The patient continued anti-TB therapy and was followed up in the outpatient unit. In the following 2 months, the patient had three admissions to hospital for episodes of pneumonia and recovered after antibiotic treatment each time. A repeat chest CT scan was performed to rule out relapse or complications of TB at 5 months of age, and revealed uniform rim calcification of multiple enlarged lymph nodes in the mediastinum, and dense mass-like consolidation of the bilateral lower lobe of the lungs with multiple fine spots of calcification (Figure 2A and B). He was weaned off home oxygen therapy at 7 months of age. The patient had anti-TB therapy according to the recommendation by Patel et al. [4], with four drugs, namely oral isoniazid (15 mg/kg/day), oral rifampin (10 mg/kg/day), oral pyrazinamide (30 mg/kg/day), and intramuscular streptomycin (20 mg/kg/day), for 2 months, then isoniazid and rifampin for an additional 6 months. He was nearly free of symptoms when anti-TB therapy was stopped. Serial follow-up radiographs were performed and showed opacities in the bilateral lung remaining at 12 months of age (Figure 3A). Nearly complete resolution of opacities with residual extensive spots of calcification were seen at 15 months of age (Figure 3B). He had attained normal growth and was normal according to the Denver developmental examination by the age of 15 months.

## Discussion

The criteria for the diagnosis of congenital TB were established by Beizke in 1935, and later revised by Cantwell et al. [5] in 1994 to increase sensitivity. According to the revised criteria, the infant must have a proven tuberculous lesion and at least one of the following: 1) lesions in the first week of life; 2) presence of a primary hepatic complex or hepatic granulomas; 3) tuberculous infection of the placenta or maternal genital tract; and 4) exclusion of postnatal exposure by contact investigation. Our case met the revised criteria.

This is the first case in China of congenital TB in an infant after *in vitro* fertilization. Congenital TB after *in vitro* fertilization is an exceedingly rare occurrence. Around 10 cases have been reported in the literature worldwide to date. With *in vitro* fertilization technology, women infertile as a result of genital TB may conceive and give birth to children with congenital TB. We suggested that the mother consult a gynecologist for evaluation of possible genital tract TB after TB in her infant was confirmed. The mother refused an endometrial biopsy. However, infertility in the infant’s mother arising from genital tract TB was highly suspected because postnatal infection of the infant was ruled out by thorough contact investigations. Our case highlights the importance of exclusion of TB in women from an epidemic area before proceeding to *in vitro* fertilization.

The symptoms of congenital TB usually present at 2–3 weeks after birth. The presenting symptoms are often non-specific, such as fever, respiratory distress, hepatosplenomegaly and abdominal distension, and often mimic common neonatal diseases [5-7]. Most clinicians are unfamiliar with congenital TB, as suggested by our case; the patient was initially misdiagnosed with sepsis or pneumonia because of the non-specific symptoms and lack of signs of active TB in his mother. TB was suspected only when the condition of the patient deteriorated after treatment with potent broad-spectrum antibiotics. In view of the unreliability of the TST, our patient underwent the T-SPOT.TB assay, with a positive result. On the basis of this result, anti-TB therapy was started on hospital day 49. Fortunately, the patient survived in spite of the delay in diagnosis.

To our knowledge, this is the first report on using the T-SPOT.TB assay for the detection of congenital TB. The T-SPOT.TB assay is one type of commercially available interferon-gamma (IFN-γ) released assays (IGRAs), which are based on IFN-γ release from specific T cells *in vitro* in response to the stimulation of MTB antigens. The T-SPOT.TB assay is an ELISPOT assay that counts individual antigen-specific T cells. Another type of commercially available IGRA is a whole blood-based test for quantification of IFN-γ released by antigen-specific T cells, and includes the QuantiFeron TB Gold and QuantiFeron TB Gold In-Tube tests. The MTB antigens used for stimulation are proteins encoded by the genomic segment of RD1 that is present in the MTB complex, and include ESAT-6 and CFP-10, which are absent in the BCG vaccine and the majority of environmental mycobacteria [8,9]. IGRAs have been used to detect TB in adults, and the T-SPOT.TB assay proved to have greater sensitivity than TSTs. Furthermore, the T-SPOT.TB assay appears to have greater specificity than TSTs in BCG-vaccinated individuals [10,11].

Unlike adults, there are few published reports on the performance of IGRAs in neonates and infants. Their reliability have not been extensively studied and validated in this age group. There are controversies about this issue because neonates and infants have different immune responses compared with adults. Decreased IFN-γ production in response to stimulation was noted in early childhood [12]. It was reported that the T-SPOT.TB assay had low sensitivity for evaluating TB in children younger than 1 year old [13]. However, some previous studies supported the applicability of IGRAs among this age group. In one study, Critselis et al. [14] reported that younger children were able to produce higher concentrations of IFN-γ in response to TB-specific antigens. In another study, Richeldi et al. [15] investigated MTB transmission using ELISPOT assays, and proved that these assays have an advantage over TSTs in the detection of MTB infection in newborns. Among these studies, data are lacking on the use of T-SPOT.TB assay in infants with congenital TB. Our report suggests that IGRAs may be a potentially useful tool in the evaluation of congenital TB. Our result was supported by a study from Tom Connell et al [16], who reported early detection of two cases of perinatal TB by QuantiFeron TB Gold, and showed that a whole blood IFN-γ release assay may be a promising tool for the diagnosis of perinatal TB.

In contrast, Luca Richield et al. [17] reported a case of latent TB infection (LTBI) acquired in the neonatal period, in which the initial ELISPOT assay at 11 weeks of age was negative. A repeat ELISPOT assay became positive when the infant was 6 months old, while the TST remained negative, and the patient finally developed active TB at 18 months of age despite chemoprophylaxis. They considered that the reason for the initial negative ELISPOT assay result may be attributed to a low mycobacteria load, because the antigen-specific T cell count is closely related to pathogen burden. Similarly, a study by Herrmann et al. [18] demonstrated that children with active TB had significantly higher IFN-γ values compared with LTBI children. In addition, a reduction in IFN-γ during anti-TB treatment in LTBI and active TB were noted [18,19]. This argued for the applicability of IGRAs because most infants with congenital TB have a high pathogen burden. It was reported that gastric aspirate samples were positive in culture in as many as 80% of cases (21).

Although developed as an alternative to TSTs, a positive IGRA result in itself indicates MTB sensitization, rather than confirmation of active TB. Nevertheless, RD1 is present in the MTB complex (*M. africanum*, *M. bovis*, *M.canettii*, *M. caprae*, *M. microti*, *M. pinnipedii*, *M. mungi* and *M. orygis*) rather than MTB [20], and sensitization to a few non-MTB species such as *M. kansasii*, *M. marinum*, *M. riyadhense* and *M. szulgai* may cause false-positive IGRA results [21,22].

Biomarkers in the blood that can distinguish between active and latent TB rapidly are unavailable to date. Other tests such as new culture-based methods, bacteriophage-based assays, and nucleic acid amplification techniques (NAAT) are being developed for the rapid detection of MTB, among which NAAT appear to be a promising test with high sensitivity [23]. These tests may play a supplementary role in the diagnosis of congenital TB in the future.

Microbiological confirmation of TB often takes several weeks and may not be available in areas with poor resources. A chest radiograph may provide signs suggesting TB, and thus initiate more extensive examination to make an early diagnosis. However, it is not uncommon for infants with congenital TB to present with non-specific imaging findings [6]. In our case, initial chest radiographs showed non-specific bilateral interstitial and alveolar infiltrates consistent with pneumonia, while the chest CT performed 12 days later revealed multiple pulmonary nodules. Chest CT scans may have an advantage over chest radiographs in detecting lymphadenopathy and parenchymal lesions, especially when chest radiographs give inconclusive or negative results. Among reports of infants with congenital TB, only a few underwent chest CT. Two cases of congenital TB were reported by Albert Chen et al [24]. They first described pulmonary nodules with peripheral rim enhancement, and a central hypodense area was a prominent imaging feature in congenital TB. Our case also suggested that pulmonary nodules may be a specific imaging feature of congenital TB, which is compatible with TB as a granulomatous disease. As evidenced in our case, the initial thoracic CT demonstrated prominent multiple pulmonary nodules, which merited sufficient attention and extensive examination for TB to make an early diagnosis. However, this finding was unnoticed at the time, possibly because both radiologists and clinicians were unfamiliar with such imaging features, given that congenital TB is a rare disease, and its imaging findings in chest CT have only been sporadically reported.

Delayed diagnosis of congenital TB often results in a fatal outcome. However, congenital TB is a curable disease, and if treated in a timely manner, remission of clinical manifestation often occurs in 2 weeks in most of patients [6]. Oxygen dependence and recurrent pneumonia in our patient may be attributable to severe lung lesions resulting from delayed diagnosis of congenital TB. The repeated chest CT revealed dense mass-like consolidation remaining at 5 months of age, despite nearly 3 months of anti-TB therapy. We considered that this long-term lesion of the lung compromised lung function and may thus have been a predisposing factor for recurrent pneumonia.

Uniform rim calcifications of multiple enlarged lymph nodes with spared central areas in the mediastinum were also noted in the repeat chest CT image. To our knowledge, such a pattern of calcification in lymph nodes has never been described in reports of congenital TB. Calcified lymph nodes and calcifications within the consolidation were both seen in our case after 3 months of anti-TB therapy. Woo Sun Kim et al. considered the presence of calcification as a diagnostic clue for TB. In their study, calcification of the lymph nodes was an uncommon imaging finding in infants with TB. It often occurred in patients who had received anti-TB therapy for 4–9 months, while calcification within the consolidation was seen in patients who had a history of anti-TB therapy for more than 6 months [25]. However, no characteristic distribution of calcification in a particular region of the lymph nodes was described in the study. Compared with that report, calcified lymph nodes and calcifications within the consolidation in our case were seen earlier. It is uncertain whether rim calcifications of lymph nodes are specific image findings for congenital TB.

Radiographic regression of the lung lesion in congenital TB was a slow process in our case. Few previous cases of congenital TB underwent repeated follow-up radiography. Complete radiographic regression of lung lesions was noted within 6 months or 12 months after treatment in two reports [26,27]. Regression of radiographic findings took longer in our case. Nearly complete resolution of opacity with remaining extensive spots of calcification occurred at 15 months of age. Such slow regression was consistent with slow remission of symptoms in our case. Nevertheless, the patient still made a complete recovery without apparent sequelae to date.

In conclusion, early identification of infants with congenital TB remains a challenge because of the non-specific symptoms and radiographic findings. The T-SPOT.TB assay may have a potential role in the early detection of congenital TB, but its results should always be considered in a clinical context. Congenital TB should be included in the differential diagnosis of infants with pneumonia that is unresponsive to potent antibiotics. Multiple pulmonary nodules in chest CT may be a clue to aid prompt diagnosis. A delay in the diagnosis of congenital TB may result in a severe lung lesion, leading to prolonged oxygen dependence, predisposition to recurrent pneumonia, and slow radiographic regression.

## Consent

Written informed consent was obtained from the patient’s parent for the publication of this report and any accompanying images.
